# Profile of Unidentified Dead Bodies in Northern India: A Tertiary Care Center Experience

**DOI:** 10.7759/cureus.104760

**Published:** 2026-03-06

**Authors:** R Sivasankary, Prashant Passi, Arushi Verma, Anubhuti Tyagi, Ramit Sai, Raviprakash Meshram, Shailesh V Parate, Ashish R Bhute

**Affiliations:** 1 Department of Forensic Medicine and Toxicology, All India Institute of Medical Sciences, Rishikesh, IND

**Keywords:** autopsy profiling, cause of death, decomposition, identification, unidentified bodies, unknown bodies

## Abstract

Introduction: Identification is the primary step in any medicolegal investigation. Unidentified bodies pose significant challenges for forensic experts and investigating officers due to the lack of history and frequent decomposition. Establishing identity is crucial for legal resolution and providing closure to families.

Aim: The aim of this study is to profile unidentified dead bodies to observe the overall patterns and characteristics of such cases in Uttarakhand.

Methods: A retrospective, record-based analysis was conducted at the Department of Forensic Medicine and Toxicology, All India Institute of Medical Sciences, Rishikesh, India. The study reviewed 633 autopsies of unidentified or unclaimed bodies performed over a six-and-a-half-year period (from January 1, 2019, to June 30, 2025). Data were analyzed using IBM Statistical Package for the Social Sciences Statistics for Windows, version 23.0 (Released 2015; IBM Corp., Armonk, NY).

Results: Unidentified bodies accounted for 15.3% (633 out of 4,132) of all autopsies. The majority were male bodies in the 21-60 year age group. Regarding clinical status, 56.24% were brought dead, and 56.39% had no history of hospital admission, while 42.65% were brought alive and 42.81% received hospital care before death. Frequent histories included natural diseases, recovery from water, or being found dead in public spaces. Approximately half of the bodies were decomposed at the time of autopsy. The leading causes of death were drowning, sepsis, bronchopneumonia, traumatic brain injury, and coronary artery disease. While forensic samples were preserved in all cases, advanced decomposition often limited definitive identification.

Conclusion: To ensure dignity and closure for families, the government should strengthen identification protocols. This requires better coordination between investigative agencies and forensic experts, along with the effective use of advanced forensic resources to reduce the number of unresolved cases.

## Introduction

Every year, millions of people across the world die or go missing without being identified, and as a result, their remains are never returned to their families or communities, leaving loved ones without closure and creating significant humanitarian, legal, and social challenges [1]. Unidentified deaths have often been described as a “silent mass disaster” [2] or a “humanitarian crisis” [3]; however, reliable data on the number of unidentified bodies and their demographic characteristics have not been systematically studied. As a result, the true extent of the problem remains poorly recognized. According to the National Crime Records Bureau (NCRB) data updated in 2016, a total of 32,708 cases were reported across India. Maharashtra recorded the highest number of cases (8,140), while Manipur and Sikkim reported the fewest, with no cases recorded [4]. Different regions have different numbers of unidentified bodies, which might be due to cultural factors and demographic variation [5]. Tourist and devotional places often report a higher number of unidentified bodies, largely due to the heavy influx of visitors from different regions, lack of local identity documents, and the transient nature of the population.

## Materials and methods

This record-based retrospective study was conducted at the Department of Forensic Medicine and Toxicology, All India Institute of Medical Sciences (AIIMS), Rishikesh, a prominent tertiary care institute in North India providing healthcare services to the surrounding population. During the study period from January 1, 2019, to June 30, 2025, a total of 4,132 medicolegal autopsies were performed, of which 633 cases (15.3%) were unknown or unidentified.

Inclusion and exclusion criteria

The study included all unknown or unidentified deceased individuals received for autopsy during the study period. This comprised whole bodies as well as partial human remains, including dismembered body parts and skeletonized remains. Cases involving identified deceased individuals were excluded.

Data collection and analysis

The primary data sources were postmortem examination reports and inquest papers received from police authorities. When available, relevant hospital records from the institution where the deceased was last treated were also reviewed. Data were compiled using Microsoft Excel (Microsoft Corporation, Redmond, WA) and subsequently analyzed with IBM Statistical Package for the Social Sciences Statistics for Windows, version 23.0 (Released 2015; IBM Corp., Armonk, NY). The findings were compared with results from other national and international studies to identify epidemiological patterns and factors contributing to unidentified deaths.

Ethical considerations

Ethical clearance was obtained from the Institutional Ethics Committee of AIIMS, Rishikesh (approval no. AIIMS/IEC/25/653). As the study involved deceased individuals and relied exclusively on official medicolegal records, the requirement for informed consent was waived in accordance with national ethical guidelines on research involving the dead.

## Results

In the present study, a total of 4,132 autopsies were conducted, of which 633 (15.3%) involved unidentified or unknown individuals. The yearly distribution showed fluctuations in the proportion of such cases, with the highest percentage observed in 2021 (19.0%), followed by 2023 (17.1%) and 2019 (16.0%). In 2024 and 2022, the proportions were 15.3% and 14.3%, respectively, while the lowest percentages were recorded in 2025 (10.3%) and 2020 (11.9%). Overall, unidentified cases consistently contributed a significant share of total autopsies, averaging approximately 15% across the study period, with notable year-to-year variations, as shown in Table 1.

**Table 1:** Year-wise distribution of total autopsies and unidentified cases (2019-2025) (n = 4,132 and 633) Study period: January 1, 2019, to June 30, 2025. Total number of autopsies done, n = 4,132. Study population: unidentified bodies, n = 633

Sr. no.	Year	Total number of unknown cases performed	Total number of cases autopsy done	Percentage
1	2019	70	435	16.0%
2	2020	42	353	11.9%
3	2021	96	505	19.0%
4	2022	98	682	14.3%
5	2023	149	870	17.1%
6	2024	138	902	15.3%
7	2025	40	385	10.3%
Total cases		633	4,132	15.3%

Age-wise distribution in Table 2 shows the highest proportion in the 31-40 years age group (149 cases, 23.5%), followed by 51-60 years (130 cases, 20.5%) and 41-50 years (116 cases, 18.3%). The 21-30 years group accounted for 78 cases (12.3%), while the 60-70 years group contributed 84 cases (13.3%). Fewer cases were observed in the extremes of age, including 20 cases (3.2%) from embryo to delivery, six cases (0.9%) from day 1 to 10 years, and seven cases (1.1%) from 11-20 years. In the elderly, 23 cases (3.6%) were between 71 and 80 years, and five cases (0.8%) were over 81 years. Additionally, the age of 15 cases (2.4%) could not be determined.

**Table 2:** Age-wise distribution of unidentified bodies (n = 633) The inability to determine the age of 15 individuals (2.4%) among unknown cases is attributed to advanced decomposition or significant mutilation, which destroyed critical dental and skeletal markers

Age	Number of unknowns	Percentage
Embryo to delivery	20	3.2%
1 day to 10 years	6	0.9%
11-20 years	7	1.1%
21-30 years	78	12.3%
31-40 years	149	23.5%
41-50 years	116	18.3%
51-60 years	130	20.5%
60-70 years	84	13.3%
71-80 years	23	3.6%
>81 years	5	0.8%
Unknown	15	2.4%
Total cases	633	-

Table 3 outlines that out of 633 cases, most were men (567, about 89.5%). Women made up 58 cases (around 9.1%), and in eight cases (just over 1.2%), the sex could not be identified.

**Table 3:** Sex-wise distribution of unidentified bodies (n = 633)

Sex	Number	Percentage (total = 633)
Male	567	89.5%
Female	58	9.1%
Unknown	8	1.2%

Out of the 633 cases, Table 4 presents that 356 individuals (56.2%) were brought dead, while 270 (42.6%) were brought alive. In seven cases (1.1%), the arrival status could not be ascertained.

**Table 4:** Arrival status of unidentified bodies (brought dead/brought alive) (n = 633) Brought alive: alive on arrival, received treatment, and died after admission in hospital Brought-in-dead or dead-before-arrival: persons who were evaluated by a physician to have no sign of life, at the time of presentation to a health facility Arrival status was classified as unknown in some cases owing to inadequate documentation, incomplete inquest papers, and absence of reliable scene details, as the police were unable to determine the exact circumstances under which the individual was found

Arrival status	Number of cases	%Distribution
Brought dead	356	56.2%
Brought alive	270	42.6%
Unknown	7	1.1%

Based on their available history at the time of examination, Table 5 describes the disease condition, noted in 186 cases (29.3%), followed by cases in which the body was recovered from water, found floating in a river, or discovered on the riverside (148 cases, 23.3%). A history of being found dead without further details was reported in 115 cases (18.1%). Trauma-related events were also significant contributors. Road traffic accidents (RTA) were documented in 77 cases (12.1%), while railway track accidents accounted for 14 cases (2.2%). Hanging was observed in 17 cases (2.6%), falls from height in eight cases (1.2%), and falls from moving trains in two cases (0.3%). Unusual circumstances included animal attacks (four cases, 0.6%), burn injuries (one case, 0.1%), physical assault (three cases, 0.4%), and recovery of only body parts (two cases, 0.3%). A small proportion of cases were attributed to poisoning (two cases, 0.3%) and skeletal remains recovery (four cases, 0.6%). In 50 cases (7.9%), the history was unknown.

**Table 5:** History available at the time of autopsy in unidentified cases (n = 633) RTA: road traffic accident

History	Number of cases	Percentage
History of being found dead	115	18.1%
Body recovered from water/floating in the river/body found on the riverside	148	23.3%
Medical condition	186	29.3%
History of skeletal remains recovery	4	0.6%
Poisoning	2	0.3%
RTA	77	12.1%
Unknown	50	7.9%
Railway track accident	14	2.2%
Hanging	17	2.6%
Fall from a moving train	2	0.3%
Animal attack	4	0.6%
Fall from height	8	1.2%
Burn	1	0.1%
Physical assault	3	0.1%
Body parts	2	0.4%
Total	633	-

Of the total 633 cases, 357 (56.3%) individuals were not admitted to the hospital, while 271 (42.8%) had a history of hospital admission. In five cases (0.7%), the hospital record or admission status was unknown, as shown in Table 6.

**Table 6:** Hospital admission status of unidentified bodies (total cases = 633)

Hospital record	Number of cases	%Distribution (total = 633)
Hospital admitted	271	42.8%
Hospital not admitted	357	56.3%
Unknown history	5	0.7%

Table 7 shows that 320 (50.5%) were in varying stages of decomposition, while 313 cases (49.4%) were fresh (nondecomposed) at the time of autopsy.

**Table 7:** Decomposition status of unidentified bodies at autopsy (n = 633)

Decomposition status	Total number of cases	Percentage
Decomposed bodies at autopsy	320	50.5%
Fresh (nondecomposed) bodies at autopsy	313	49.4%

Tables 8, 9 show the classification of deaths and their causes. Of the 633 cases studied, natural deaths accounted for 324 cases (51.18%), with the most common causes being sepsis (101 cases, 15.9%), bronchopneumonia (89 cases, 14.1%), and pulmonary tuberculosis (37 cases, 5.8%). Other natural causes included coronary artery disease, chronic liver disease, chronic obstructive pulmonary disease, interstitial lung disease, pulmonary edema, hypertrophic cardiomyopathy, cerebrovascular accidents, intracerebral hemorrhage, starvation, and carcinomas, contributing to smaller proportions of cases. Unnatural deaths comprised 232 cases (36.6%), with leading causes being antemortem drowning (102 cases, 16.1%), traumatic brain injury (65 cases, 10.3%), and hemorrhagic shock due to trauma (37 cases, 5.8%). Other causes included hanging/strangulation, spine injury, burn injury, crush injury, decapitation, animal bite, hypothermia, and poisoning. Additionally, fetal deaths and cases where the cause could not be ascertained accounted for 77 cases (12.2%).

**Table 8:** Classification of death as natural, unnatural, and undetermined (total cases = 633) The undetermined category included fetal deaths and other cases in which the cause of death could not be ascertained

Classification of causes of death in autopsied cases	Total number of cases	Percentage
Natural death	324	51.18%
Unnatural death	232	36.6%
Undetermined	77	12.16%

**Table 9:** Cause of death in unidentified bodies (total cases = 633) Sepsis unspecified is an accepted ICD-10-based cause of death used when sepsis is established, but the precise source or etiological agent cannot be identified. It is distinct from the cause of death, which could not be ascertained due to the absence of conclusive anatomical, pathological, or toxicological findings, and lack of adequate history or circumstantial evidence ICD-10: International Classification of Diseases, Tenth Revision

Sr. no.	Cause of death	Total number of cases	Percentage
1	Sepsis unspecified	90	14.2%
2	Sepsis (specific causes): infected wound (two), acute gastroenteritis (one), empyema (two), lower limb cellulitis (one), perforation peritonitis (three), necrotizing tubercular pericarditis (one), meningitis (one)	11	1.7%
3	Bronchopneumonia	89	14.1%
4	Pulmonary tuberculosis	37	5.8%
5	Chronic obstructive pulmonary disease	9	1.4%
6	Interstitial lung disease	5	0.8%
7	Pulmonary edema	2	0.3%
8	Asphyxia due to aspiration	8	1.3%
9	Coronary artery disease	42	6.6%
10	Chronic liver disease	9	6.6%
11	Hypertrophic cardiomyopathy	3	0.5%
12	Intracerebral hemorrhage	6	0.9%
13	Cerebrovascular accident	2	0.3%
14	Traumatic brain injury	65	10.3%
15	Spine injury	2	0.3%
16	Crush injury	1	0.2%
17	Decapitation	1	0.2%
18	Burn injury	2	0.3%
19	Hemorrhagic shock due to trauma	37	5.8%
20	Antemortem drowning	102	16.1%
21	Antemortem hanging	17	2.7%
22	Strangulation	1	0.2%
23	Starvation	3	0.5%
24	Animal bite	4	0.6%
25	Hypothermia	1	0.2%
26	Carcinoma (gall bladder, lung, liver)	5	0.8%
27	Poisoning	2	0.3%
28	Fetal death cause unspecified	20	3.2%
29	Could not be ascertained	57	9%
Total		633	-

For DNA-based identification (Table 10), molar teeth were the most frequently preserved samples (558 cases), followed by sternum (44 cases), premolar teeth (18 cases), blood on gauze (10 cases), femur bone (two cases), and tibial bone (one case). Fingerprints were obtained in all cases wherever feasible. In addition to DNA samples, case-based ancillary samples were collected and forwarded to the Forensic Science Laboratory depending on the circumstances of death. These included the stomach and small intestine with contents; parts of the liver and spleen, and half of each kidney; 10 mL of blood preserved in five sodium fluoride-containing vials (2 mL each); and a control solution of saturated sodium chloride, in 46 cases. Other ancillary samples comprised scalp hair (seven cases), vaginal swabs (three cases), muscle tissue (four cases), nail clippings (five cases), ligature material (eight cases), clothes (eight cases), whole fetus (two cases), maggots (one case), and mesenteric lymph nodes (one case).

**Table 10:** Samples preserved in unidentified cases In addition to samples preserved for DNA analysis, ancillary samples were sent to the Forensic Science Laboratory and for histopathological examination depending on case circumstances, including viscera and blood with control solution (46 cases), scalp hair (seven), vaginal swabs (three), maggots (one), muscle (four), nail clippings (five), whole fetus (two), ligature material (eight), clothes (eight), and mesenteric lymph nodes (one)

Samples preserved	Number of cases
Molar tooth (for DNA analysis)	558
Premolar tooth (for DNA analysis)	18
Sternum (for DNA analysis)	44
Blood on gauze (for DNA analysis)	10
Femur bone (for DNA analysis)	02
Tibial bone (for DNA analysis)	01
Finger print	627
Ancillary samples collected	85

## Discussion

Identification is essential for legal purposes and applies to both living and deceased persons. Identification is the process of establishing an individual's unique characteristics; it may be complete when identity is conclusively confirmed or partial when only certain features, such as age, sex, race, or other distinguishing characteristics, are determined [6]. An unknown body refers to a deceased individual who lacks a legal representative or identifiable next of kin to arrange final disposition [6]. Although Article 21 [7] does not explicitly address rights after death, the Supreme Court in Parmanand Katara v. Union of India held that the right to dignity extends to the deceased, requiring respectful handling of dead bodies. This was reaffirmed in Ashray Adhikar Abhiyan v. Union of India, which recognized the State’s duty to ensure dignified burial or cremation of even unclaimed and homeless deceased persons in accordance with their faith and customs [7].

In India, the management of unidentified bodies is governed by the Code of Criminal Procedure, now replaced by Section 194 of the Bharatiya Nagarik Suraksha Sanhita (BNSS), supplemented by guidelines issued by the National Human Rights Commission (NHRC) and provisions in State Police Manuals. As per standard practice, an unidentified body is preserved in a mortuary for 72 hours to allow relatives or acquaintances to claim it, with the period extendable up to 7-10 days if there are leads regarding identity and adequate mortuary facilities. The case is treated as a medicolegal case, and an autopsy is conducted only after completion of a police or magisterial inquest under Section 194 BNSS. During the postmortem examination, all identifying features such as scars, tattoos, deformities, and clothing are meticulously documented; fingerprints are mandatorily taken, DNA samples are preserved for future identification, dental charting is carried out when indicated, and frontal facial photographs are taken, with videography advised in suspicious cases in line with NHRC guidelines. Simultaneously, the investigating officer circulates a hue and cry notice, cross-checks details with missing persons records through the NCRB and the Zonal Integrated Police Network (ZIPNET) database, publishes photographs and descriptions in the media, and displays personal effects to facilitate identification. If the body remains unclaimed after the stipulated period and the autopsy is complete, the police assume legal responsibility for final disposal, coordinating with municipal authorities and, frequently, nongovernmental organizations to perform cremation or burial in accordance with inferred religious practices, after obtaining a medical no-objection certificate and ensuring that all identification records, including fingerprints and DNA, have been duly preserved [8,9].

In our study, a total of 4,132 autopsies were performed, of which 633 cases (15.32%) involved unidentified individuals. Compared with other studies, the proportion of unidentified cases varied across regions and study periods, as detailed in Table 11, with reported rates ranging from 10.55% to 13.9% in studies from Srinagar (Uttarakhand) [10], Lucknow (Uttar Pradesh) [11], and BG Nagara (Karnataka) [12]. This substantial proportion reflects a significant medicolegal and logistical burden on the institution due to prolonged body preservation and delayed investigative processes. Our higher burden may be partly attributed to the presence of major devotional tourist destinations in the region. Such areas attract large transient populations, including pilgrims and migrants, increasing the likelihood of deaths occurring away from home and without valid identification, which highlights the need for robust identification mechanisms in pilgrimage-heavy regions.

**Table 11:** Comparison with different studies

Study location	Period	Total autopsy	Unidentified cases	Reference
Rishikesh, Uttarakhand	2019-June 2025	4,132	633 (15.32%)	Present study
Srinagar, Uttarakhand	2018-2022	407	56 (13.5%)	[10]
Lucknow, Uttar Pradesh	2004-2013	39,872	5,542 (13.9%)	[11]
BG Nagara, Karnataka	2021-2024	796	84 (10.55%)	[12]

In the present study, the predominant age group among unidentified bodies was 21-30 years, with 75% of cases occurring between 21 and 60 years, indicating that unidentified deaths largely affect the economically productive population. A similar age distribution was reported by Baied in a seven-year study from South Africa, which showed a predominance of younger individuals aged 20-39 years, supporting the present findings [1]. The peak incidence observed in the 31-40-year age group (23.5%) suggests increased vulnerability among young and middle-aged adults, possibly related to migration, homelessness, occupational hazards, substance use, and limited social support systems. In contrast, studies by Singh and Basra, and Gitanjali reported a higher proportion of unidentified bodies in older age groups, namely 70-79 and 61-70 years, respectively, highlighting regional and demographic variations in the age profile of unidentified deaths [6,13].

In the current analysis, a marked male predominance was observed among unidentified bodies. A similar male predominance has been consistently reported in earlier studies by Singh et al., Gitanjali, Chikhalkar et al., Altun et al., Chattopadhyay et al., and Cheung and Hwang, with Singh et al. reporting male cases constituting 85.6%, compared to 14.4% female cases [11,13-17]. This consistent trend across studies reflects prevailing patriarchal social structures, wherein women are more likely to remain within household settings and maintain stronger familial ties, while men more frequently migrate in search of employment and economic opportunities. Consequently, men are at greater risk of dying away from their native place and family support systems. In devotional or pilgrimage centers, many such individuals live away from their families, sometimes resembling social or familial abandonment due to estrangement or neglect, with these locations perceived as refuges to spend the latter part of life. Further supporting this interpretation, Reid et al. reported that individuals with weak social connections or limited community involvement were more likely to remain unidentified after death. Poor social ties hinder tracing of family members or identification of next of kin, a challenge compounded by the high cost of travel to forensic facilities, which many economically disadvantaged families cannot afford, particularly when interstate or interprovincial travel is required due to urban migration [1].

Among 633 unidentified cases, more than half of the individuals, 356 cases (56.24%), were brought dead to the hospital, while 270 cases (42.65%) were brought alive; in seven cases (1.1%), the arrival status could not be ascertained. This predominance of brought-dead cases highlights significant delays in detection, transportation, and access to timely medical care among unidentified individuals, who often belong to socially marginalized or transient populations. The finding underscores critical gaps in prehospital emergency services and early medical intervention, suggesting that many of these deaths may have been preventable with timely recognition and healthcare access.

In the present study, disease-related conditions constituted the most common documented history (29.38%), followed by bodies recovered from water bodies (23.38%) and cases found dead without any specific antecedent history (18.17%). Trauma-related causes formed a substantial proportion, with RTAs (12.16%) being the most frequent, while railway incidents, hanging, and falls contributed smaller yet notable shares. Unusual and violent circumstances such as animal attacks, burns, assaults, poisoning, and recovery of body parts or skeletal remains were relatively infrequent. Notably, nearly 8% of cases had a completely unknown history, highlighting significant gaps in circumstantial information and the difficulty in reconstructing events leading to death in unidentified individuals. Comparable observations have been reported in earlier studies, with Singh and Basra documenting natural disease processes in 41.6% of cases, Gitanjali reporting natural causes in approximately 18.3%, and Kaur et al. observing a predominance of natural deaths accounting for 67.46%, collectively underscoring that natural diseases, often compounded by inadequate circumstantial information, remain major contributors to unidentified deaths and pose persistent humanitarian challenges [5,6,13].

More than half of the unidentified individuals, 357 cases (56.39%), had no history of hospital admission, while 271 cases (42.81%) were admitted prior to death; hospital admission status was unknown in only five cases (0.78%). This finding indicates that a substantial proportion of deaths occurred outside the formal healthcare system, reflecting delayed recognition, lack of access to medical services, or sudden death in public or isolated settings. Indian and international studies indicate that only a small proportion of patients are admitted as unknown, with an Indian study reporting approximately 1% unidentified admissions to a tertiary care trauma center and an international study showing a comparable figure of about 1.8% unidentified patients in the emergency department [18,19].

Regarding decomposition, 320 cases (50.55%) were in varying stages of decomposition, while 313 cases (49.45%) were fresh at the time of autopsy. In contrast, Mahadeva et al. reported a predominance of fresh unidentified dead bodies (UIDBs), with 70 cases (83.3%) received in a fresh state and only 14 cases (16.7%) in a decomposed condition [12]. The near-equal distribution highlights delayed discovery and recovery in a substantial proportion of unidentified deaths, often influenced by prolonged postmortem intervals and adverse environmental conditions. Advanced decomposition poses significant challenges to cause-of-death determination and personal identification, underscoring the need for timely body recovery, improved surveillance, and the use of forensic anthropology and DNA-based identification techniques in such cases.

Among the natural causes of death, 321 cases (50.7%) were due to conditions such as sepsis, bronchopneumonia, and pulmonary tuberculosis, reflecting a substantial burden of untreated infections among unidentified individuals. Unnatural deaths accounted for 232 cases (36.6%), with antemortem drowning as the most common cause, followed by traumatic brain injury and hemorrhagic shock due to trauma. Fetal deaths and cases with undetermined causes constituted 12.2%, largely attributable to advanced decomposition. Overall, these findings indicate that unidentified deaths in Indian settings are predominantly driven by preventable natural diseases, particularly infections [5,13,20]. In contrast, international studies, such as those from Izhevsk (2004-2005) and Russia (2002), have reported a predominance of unnatural causes of death, including exposure to natural cold, violence, and other external factors, underscoring regional differences in social determinants, environmental exposure, and patterns of vulnerability among unidentified populations [21].

A wide range of samples was preserved to aid identification and medicolegal evaluation in unidentified bodies. Teeth, particularly molars, were the most commonly retained DNA sources, reflecting their durability in advanced decomposition, while fingerprints were obtained in all feasible cases. In addition, case-based ancillary samples were collected and sent for forensic analysis depending on the circumstances of death, including viscera, blood with preservative, control solution, and specific materials such as hair, vaginal swabs, ligature material, clothes, maggots, and fetal remains.

Comparison of identification systems

The United States operates a comprehensive national system known as the National Missing and Unidentified Persons System (NamUs), funded by the Department of Justice. NamUs functions as a centralized online database for missing persons, unidentified human remains, and unclaimed bodies, enabling law enforcement agencies, medical examiners, coroners, and the public to search and cross-match records. In addition to database access, NamUs provides free forensic services, including DNA analysis, fingerprinting, forensic odontology, and anthropology, along with investigative assistance and professional training. This integrated, nationwide approach significantly enhances identification outcomes and interagency coordination [22].

In the Indian system, Aadhaar is often viewed as a first-line means of identification; however, its use is strictly limited to civil purposes and accessible only to authorized government agencies due to its highly confidential nature. Aadhaar is designed for identity verification rather than identity establishment and requires prior enrolment, consent, and live biometric authentication, making it unsuitable for identifying unknown or unidentified individuals in medicolegal contexts. Legal and ethical restrictions further preclude its use by civil personnel, including doctors, even for investigative purposes. Consequently, established forensic methods such as fingerprints, dental records, and DNA profiling remain the gold standard for identification in unknown cases [23,24].

Several region-specific and institutional platforms support the identification of UIDBs. ZIPNET plays a key role in northern India by facilitating information sharing among law enforcement agencies. It maintains records of over 70,000 unidentified bodies and allows police to upload basic physical details and photographs, enabling real-time access across participating states. The platform also supports the digital circulation of Hue and Cry notices, improving the speed of information dissemination. However, ZIPNET has notable limitations, including the absence of automated linkage between missing persons and UIDB records, dependence on manual searches, restricted regional coverage despite high interstate migration, and variable data quality with incomplete descriptions and poor-quality images. Currently, ZIPNET operates across eight northern states and union territories, Delhi, Haryana, Rajasthan, Uttar Pradesh, Punjab, Uttarakhand, Chandigarh, and Himachal Pradesh, highlighting the need for stronger integration with national systems such as Crime and Criminal Tracking Network and Systems [25].

At the institutional level, Behera et al. developed UMID (Unidentified Bodies and Missing Persons Identification Portal and DNA database), a web-enabled, publicly accessible platform. UMID allows users to view phenotypic, anthropological, and visual characteristics of unidentified individuals, supported by advanced search functions [26]. The system also incorporates secure storage of DNA profiles using the Combined DNA Index System core Short Tandem Repeat (STR) loci for confirmatory identification [27]. Data, including photographs, clothing descriptions, scars, tattoos, anthropological findings, and postmortem details, are uploaded after informed consent from the investigating officer, the legal custodian under Sections 174 and 176 of the Criminal Procedure Code, India. While visual identifiers aid presumptive identification, they are often unreliable in decomposed or disfigured bodies. DNA profiling using STR markers, therefore, remains the gold standard for definitive identification [27]. Ethical safeguards are ensured by storing only nonphenotypic STR loci, restricting access, and discarding claimant DNA profiles after successful matching, in line with Indian regulations and international guidelines [28-30].

Emerging forensic technologies

In addition to database-driven systems, intraoral scanners represent an emerging tool with significant forensic potential [31-33]. These devices generate accurate three-dimensional images of teeth and oral structures and, if implemented mandatorily across hospitals, analogous to the Aadhaar biometric system, could support a centralized digital oral database. Integration of intraoral scan data with fingerprints and DNA profiles would further strengthen identification processes. Three-dimensional palatal scans, including palatal rugae and anatomical landmarks, are particularly valuable in RTAs and mass disasters, as the palate is relatively well protected from damage and remains useful even in edentulous individuals where conventional dental identification is limited [33].

Limitations

This study is limited by its retrospective, single-center design and dependence on medicolegal records, which may contain incomplete clinical, circumstantial, or identification data. Further, once the postmortem report is dispatched, subsequent identification of the deceased, if any, occurs through police or judicial processes and is not routinely communicated to the certifying medical officer, precluding documentation of delayed identification outcomes. This procedural documentation typically remains confined to the police station records and is not routinely communicated back to the autopsy department.

## Conclusions

India can significantly strengthen the identification of unidentified bodies and unknown hospital admissions by integrating existing systems and technologies. ZIPNet should be expanded beyond police use and linked in real time with hospitals, forensic departments, mortuaries, and newly developed civil forensic databases such as UMID. Mandatory, standardized uploading of photographs, clothing details, scars, tattoos, anthropological findings, and hospital admission details (for unknown patients) should be ensured nationwide. Newly developed civil DNA databases should be scaled to a national level, with strict ethical safeguards, voluntary consent for relatives, and limited STR-based profiling only for identification purposes. Hospitals should be mandated to register all unknown patients and unidentified deaths on a centralized platform at the time of admission or death. The use of oral/intraoral scanners (palatal morphology) can be incorporated as an adjunct biometric tool, especially in decomposed, burnt, or fingerprint-negative cases, and linked with dental colleges and forensic odontologists. Artificial intelligence-based image matching (facial, clothing, tattoos) and advanced search filters can further improve matching efficiency. A single national portal, multilingual and publicly accessible, combining phenotypic, biometric, odontological, and DNA data, supported by legal backing, standard operating procedures, and interstate coordination, can substantially reduce the number of unidentified bodies and unknown hospital patients in India.

The role of a forensic doctor should not be limited to sample collection and forwarding specimens to the forensic science laboratory. Forensic professionals must actively participate in postanalysis processes, including data interpretation, record linkage, and follow-up matching to facilitate identification. Maintaining local and regional records on the number of unidentified individuals examined and subsequently identified can help assess outcomes and improve systems of care. Beyond technical responsibilities, forensic practice carries a fundamental humanitarian obligation. Assisting in restoring identity to the deceased and unknown patients is an essential human duty, extending beyond administrative roles, financial incentives, or professional hierarchies. Even small efforts at the individual level can contribute meaningfully to dignity, closure for families, and societal trust in forensic services.
